# Supplementary material for: ProGeM: a framework for the prioritization of candidate causal genes at molecular quantitative trait loci
Source: Nucleic Acids Res. 2018 Sep 20;47(1):e3. doi: 10.1093/nar/gky837 (PMC6326795; doi:10.1093/nar/gky837)
Supplement: Supplementary Data [file gky837_supplemental_files.zip › Stacey_Supplementary_Figures.pdf]

SUPPLEMENTARY MATERIAL

ProGeM: A framework for the prioritisation of candidate causal genes at molecular quantitative trait loci

David Stacey[1,*], Eric B. Fauman[2], Daniel Ziemek[3], Benjamin B. Sun[1], Eric L. Harshfield[1,4], Angela M. Wood[1], Adam S. Butterworth[1], Karsten Suhre[5], and Dirk S. Paul[1,*]

[1] MRC/BHF Cardiovascular Epidemiology Unit, Department of Public Health and Primary Care, University of Cambridge, Cambridge, UK
[2] Pfizer Worldwide Research & Development, Genome Sciences & Technologies, Cambridge, MA, USA
[3] Pfizer Worldwide Research & Development, Inflammation & Immunology, Berlin, Germany
[4] Department of Clinical Neurosciences, University of Cambridge, Cambridge, UK
[5] Department of Physiology and Biophysics, Weill Cornell Medicine-Qatar, Doha, Qatar

* To whom correspondence should be addressed. Tel: +44 (0)1223 747217; Email: ds763@medschl.cam.ac.uk. Correspondence may also be addressed to. Tel: +44 (0)1223 761918; Email: dsp35@medschl.cam.ac.uk.

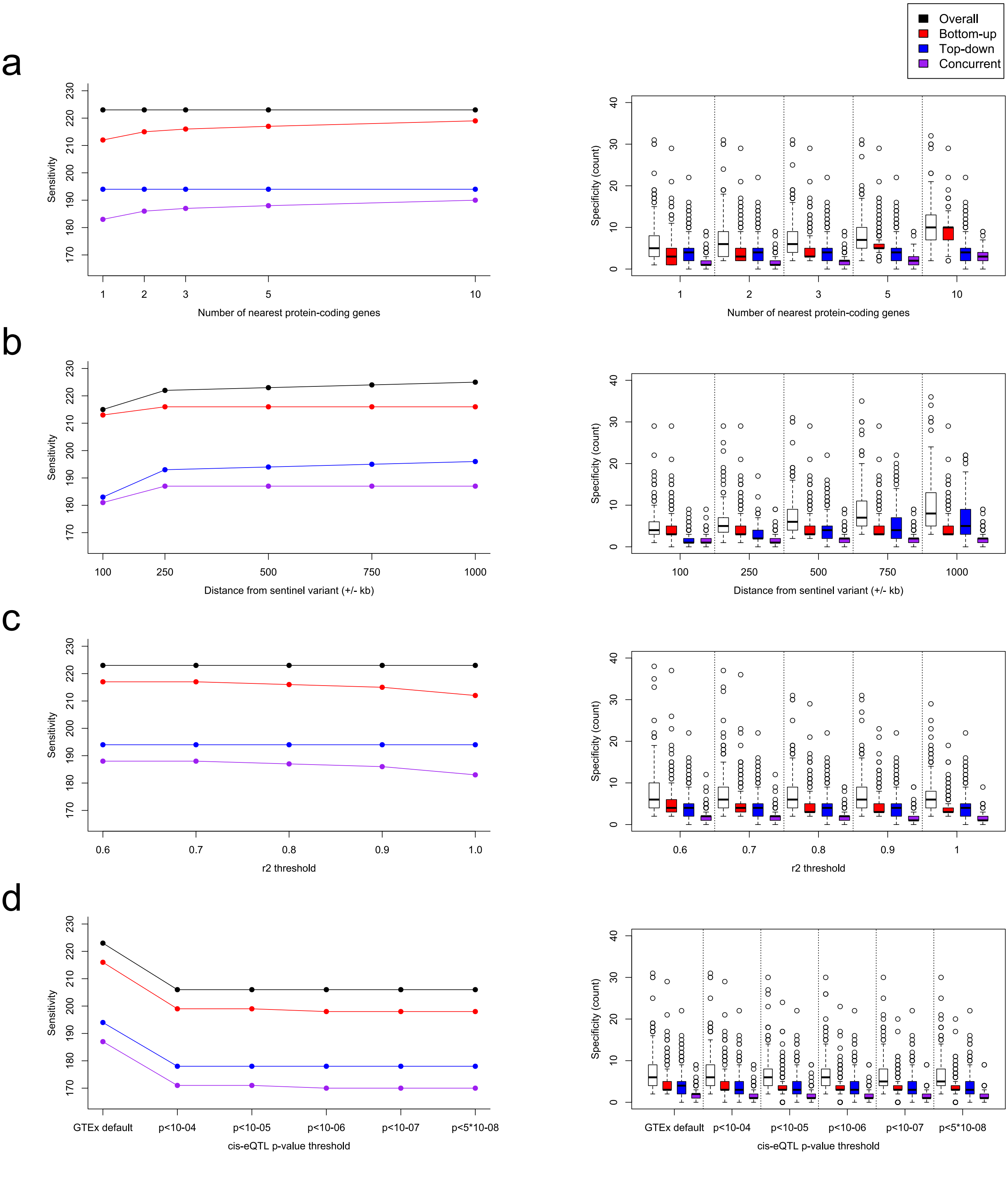

Supplementary Figure 1. The effects of varying user-defined parameter settings on the sensitivity (line graphs) and specificity (box plots) of ProGeM when applied to a dataset comprising 227 mQTLs. (**a**) Number of nearest genes to each sentinel variant (default=3); (**b**) distance window encompassing each sentinel variant from which to draw candidates (default=500kb); (**c**) r2 threshold for selecting proxies (default=0.8); and (**d**) cis-eQTL p-value threshold for selecting cis-eQTL targets as candidate causal genes (default=GTEx default threshold). The box plots show the median and interquartile ranges, with the whiskers extending to 1.5-times the corresponding interquartile range. Data points outside of this range are indicated individually as circles.

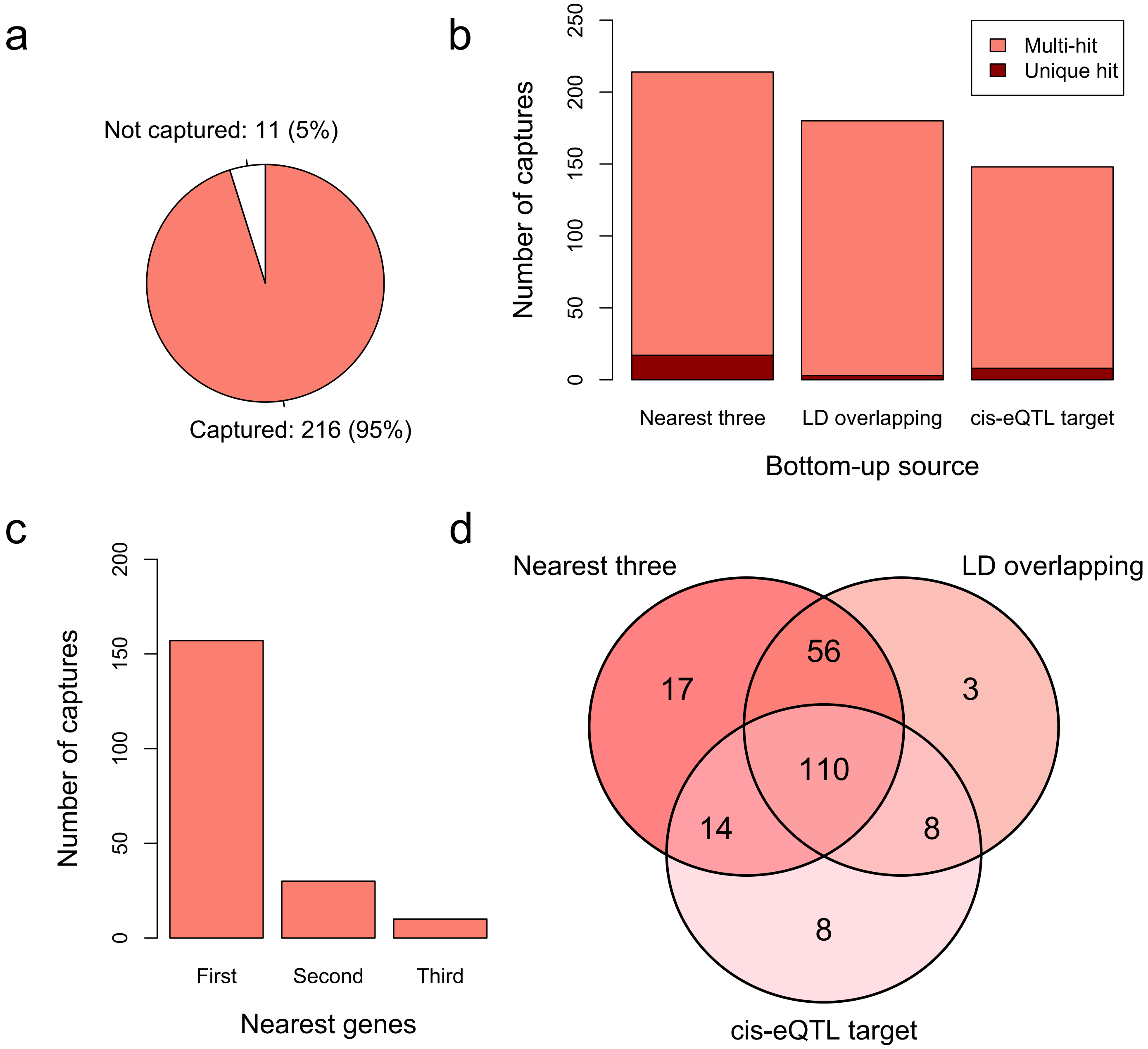

Supplementary Figure 2. Benchmarking ProGeM: bottom-up sensitivity. (**a**) Overall number (percentage) of high-confidence mQTL causal genes captured by the bottom-up component of our framework; (**b**) Number of high-confidence metabolite QTL causal genes captured by each of the three bottom-up sources utilised by our framework; (**c**) Number of captured high-confidence causal genes that were one of the three nearest protein-coding genes to their corresponding sentinel variant; (**d**) Summary of the relative contributions of the three bottom-up sources utilised by our framework towards identifying high-confidence causal genes.

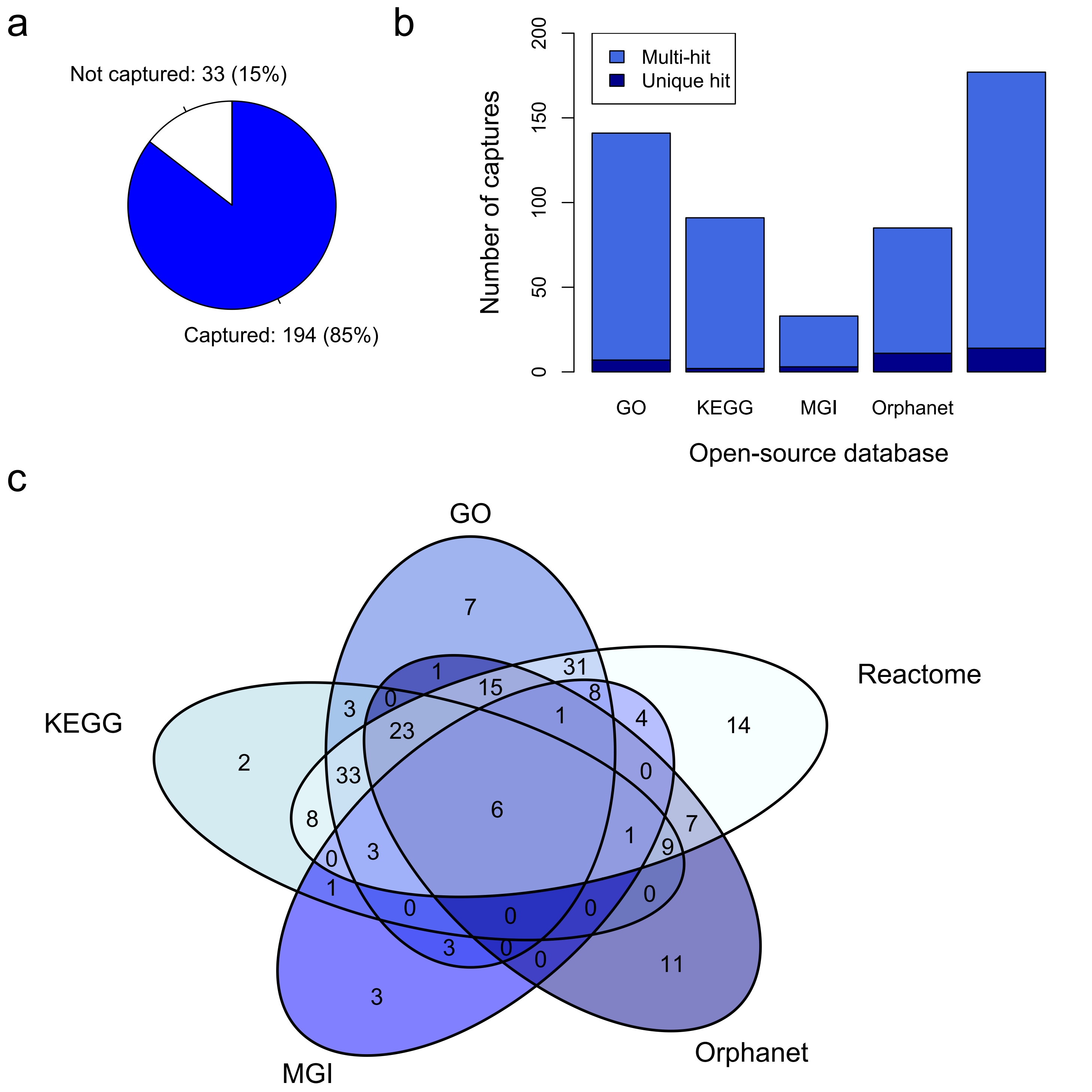

Supplementary Figure 3. Benchmarking ProGeM: top-down sensitivity. (**a**) Overall number (percentage) of high-confidence mQTL causal genes captured by the top-down component of ProGeM; (**b**) and (**c**) Summaries of the number of high-confidence mQTL causal genes captured either uniquely or concurrently by the five open-source databases utilised by the top-down component of our framework.

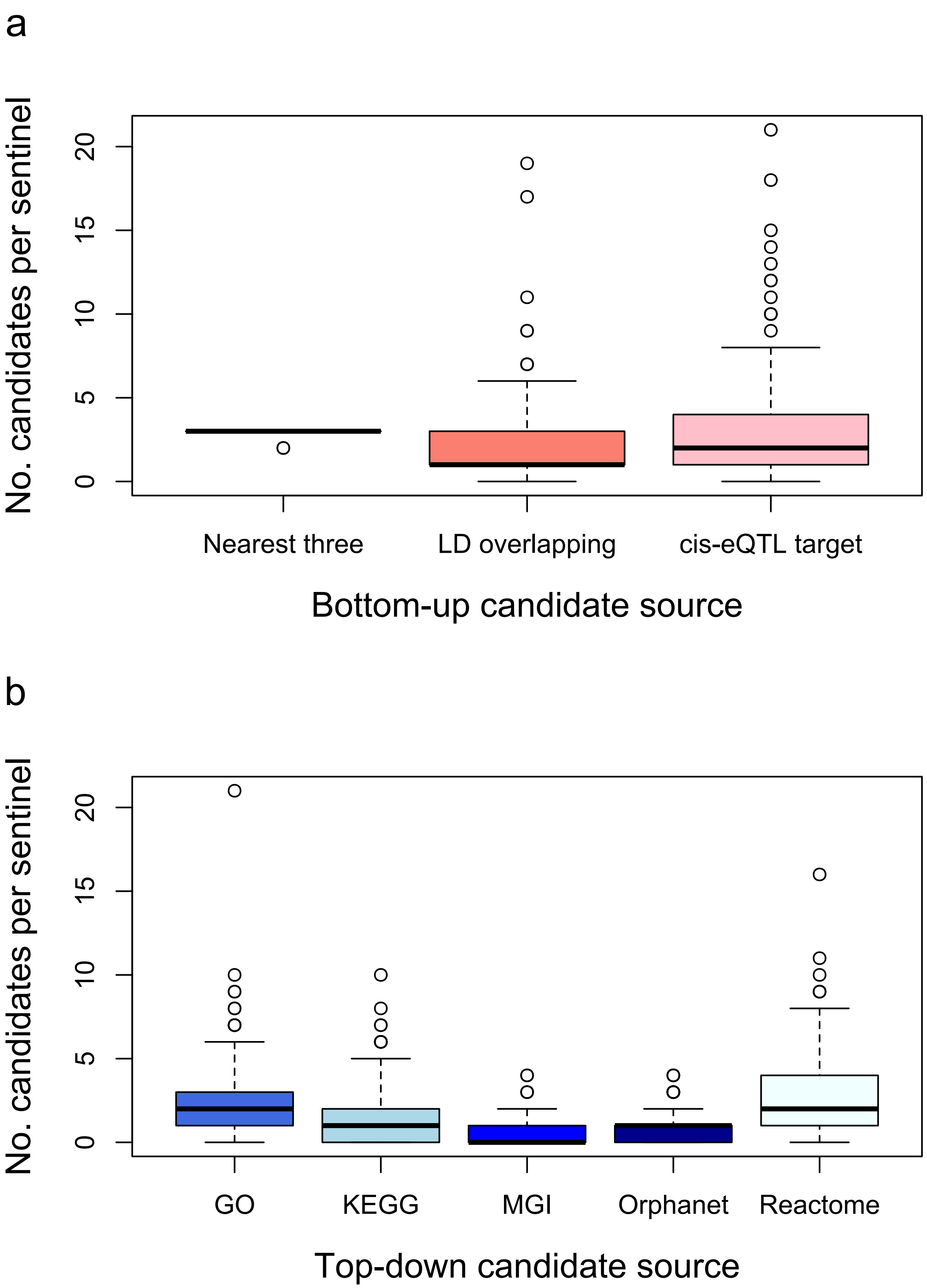

Supplementary Figure 4. Benchmarking ProGeM: bottom-up and top-down specificity. Box plots summarising the specificity ("background noise") associated with each of the data sources utilised by the (**a**) bottom-up and (**b**) top-down components of ProGeM.

a

Tissue	n	TP / FP	OR	Adj p-value
Adipose Subcutaneous	385	37 / 83	3.17	2.55e-05
Adipose Visceral Omentum	313	26 / 58	3.06	1.12e-03
Adrenal Gland	175	17 / 32	3.54	6.13e-03
Artery Aorta	267	24 / 71	2.27	8.55e-02
Artery Coronary	152	7 / 20	2.24	1.000
Artery Tibial	388	35 / 92	2.66	9.21e-04
Brain Amygdala	88	6 / 6	6.44	1.33e-01
Brain Anterior cingulate cortex BA24	109	13 / 16	5.37	1.82e-03
Brain Caudate basal ganglia	144	10 / 21	3.09	2.90e-01
Brain Cerebellar Hemisphere	125	17 / 26	4.37	8.60e-04
Brain Cerebellum	154	22 / 39	3.83	2.84e-04
Brain Cortex	136	14 / 23	4.02	8.63e-03
Brain Frontal Cortex BA9	118	10 / 15	4.35	4.54e-02
Brain Hippocampus	111	7 / 12	3.76	4.61e-01
Brain Hypothalamus	108	5 / 10	3.20	1.000
Brain Nucleus accumbens basal ganglia	130	10 / 13	5.02	2.04e-02
Brain Putamen basal ganglia	111	7 / 16	2.81	1.000
Brain Spinal cord cervical c-1	83	6 / 10	3.85	7.16e-01
Brain Substantia nigra	80	4 / 8	3.19	1.000
Breast Mammary Tissue	251	14 / 42	2.17	8.37e-01
Cells EBV-transformed lymphocytes	117	5 / 19	1.67	1.000
Cells Transformed fibroblasts	300	33 / 82	2.80	6.61e-04
Colon Sigmoid	203	21 / 39	3.64	8.07e-04
Colon Transverse	246	27 / 47	3.98	1.69e-05
Esophagus Gastroesophageal Junction	213	19 / 46	2.75	3.39e-02
Esophagus Mucosa	358	41 / 105	2.79	7.68e-05
Esophagus Muscularis	335	32 / 73	3.06	2.42e-04
Heart Atrial Appendage	264	27 / 52	3.58	7.72e-05
Heart Left Ventricle	272	28 / 56	3.46	8.66e-05
Liver	153	17 / 26	4.37	8.60e-04
Lung	383	28 / 82	2.32	3.68e-02
Minor Salivary Gland	85	4 / 7	3.65	1.000
Muscle Skeletal	491	41 / 90	3.29	1.93e-06
Nerve Tibial	361	45 / 116	2.81	1.92e-05
Ovary	122	4 / 12	2.12	1.000
Pancreas	220	20 / 45	2.98	1.31e-02
Pituitary	157	18 / 23	5.27	7.78e-05
Prostate	132	7 / 22	2.04	1.000
Skin Not Sun Exposed Suprapubic	335	35 / 76	3.25	1.98e-05
Skin Sun Exposed Lower leg	414	48 / 107	3.33	1.90e-07
Small Intestine Terminal Ileum	122	7 / 17	2.65	1.000
Spleen	146	9 / 31	1.86	1.000
Stomach	237	24 / 40	4.11	4.49e-05
Testis	225	31 / 64	3.38	6.44e-05
Thyroid	399	39 / 98	2.83	8.04e-05
Uterus	101	5 / 5	6.41	3.10e-01
Vagina	106	5 / 11	2.91	1.000
Whole Blood	369	22 / 67	2.19	1.83e-01

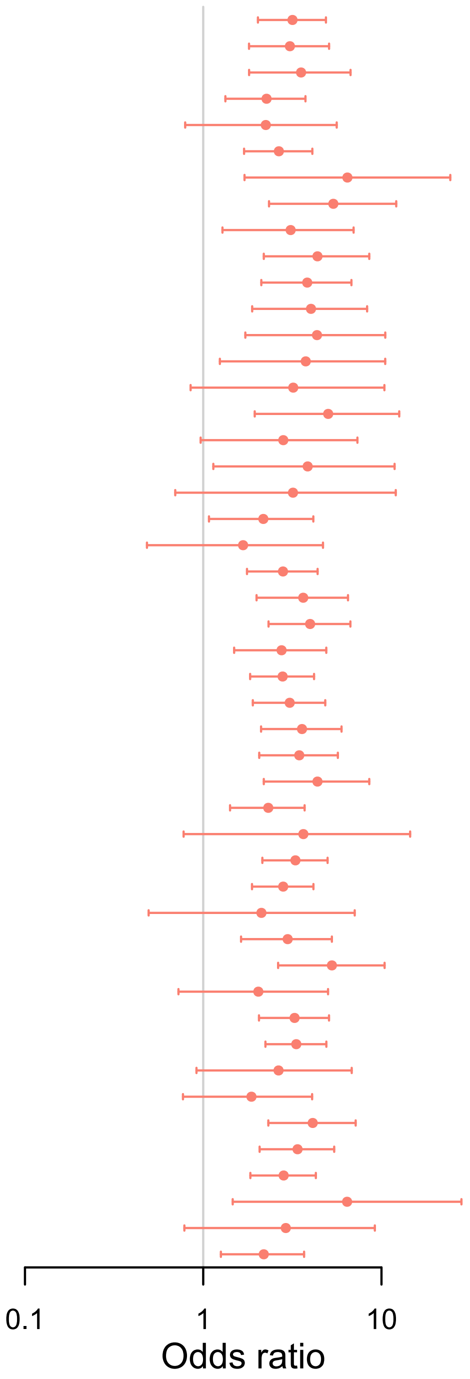

b

Tissue	n	TP / FP	OR	Adj p-value
Adipose Subcutaneous	385	43 / 100	3.12	3.39e-06
Adipose Visceral Omentum	313	30 / 65	3.20	0.000157
Adrenal Gland	175	19 / 37	3.44	0.003433
Artery Aorta	267	27 / 84	2.17	0.073364
Artery Coronary	152	12 / 28	2.80	0.374420
Artery Tibial	388	41 / 107	2.73	9.75e-05
Brain Amygdala	88	7 / 9	5.02	0.150620
Brain Anterior cingulate cortex BA24	109	14 / 18	5.16	0.001265
Brain Caudate basal ganglia	144	10 / 24	2.70	0.894255
Brain Cerebellar Hemisphere	125	21 / 35	4.07	0.000228
Brain Cerebellum	154	26 / 53	3.37	0.000276
Brain Cortex	136	18 / 30	4.02	0.001141
Brain Frontal Cortex BA9	118	11 / 20	3.59	0.081191
Brain Hippocampus	111	7 / 14	3.22	0.835097
Brain Hypothalamus	108	5 / 11	2.91	1.000
Brain Nucleus accumbens basal ganglia	130	11 / 23	3.12	0.187878
Brain Putamen basal ganglia	111	6 / 20	1.92	1.000
Brain Spinal cord cervical c-1	83	7 / 13	3.47	0.628114
Brain Substantia nigra	80	5 / 8	4.00	1.000
Breast Mammary Tissue	251	17 / 52	2.15	0.544134
Cells EBV-transformed lymphocytes	117	9 / 25	2.32	1.000
Cells Transformed fibroblasts	300	35 / 97	2.51	0.002469
Colon Sigmoid	203	26 / 50	3.57	0.000120
Colon Transverse	246	33 / 60	3.89	1.58e-06
Esophagus Gastroesophageal Junction	213	19 / 48	2.63	0.071580
Esophagus Mucosa	358	42 / 126	2.36	0.001303
Esophagus Muscularis	335	33 / 94	2.42	0.005934
Heart Atrial Appendage	264	32 / 72	3.10	0.000118
Heart Left Ventricle	272	32 / 66	3.40	4.07e-05
Liver	153	23 / 30	5.27	3.21e-06
Lung	383	32 / 93	2.36	0.009654
Minor Salivary Gland	85	5 / 10	3.20	1.000
Muscle Skeletal	491	46 / 108	3.12	2.00e-06
Nerve Tibial	361	49 / 132	2.72	2.10e-05
Ovary	122	6 / 16	2.40	1.000
Pancreas	220	23 / 60	2.58	0.022329
Pituitary	157	19 / 38	3.35	0.004526
Prostate	132	9 / 23	2.53	1.000
Skin Not Sun Exposed Suprapubic	335	40 / 87	3.31	2.45e-06
Skin Sun Exposed Lower leg	414	50 / 127	2.91	2.33e-06
Small Intestine Terminal Ileum	122	11 / 20	3.59	0.081191
Spleen	146	12 / 41	1.89	1.000
Stomach	237	33 / 53	4.43	1.56e-07
Testis	225	36 / 85	2.99	6.84e-05
Thyroid	399	47 / 121	2.83	9.17e-06
Uterus	101	6 / 10	3.85	0.716100
Vagina	106	6 / 11	3.50	0.983049
Whole Blood	369	25 / 81	2.06	0.234513

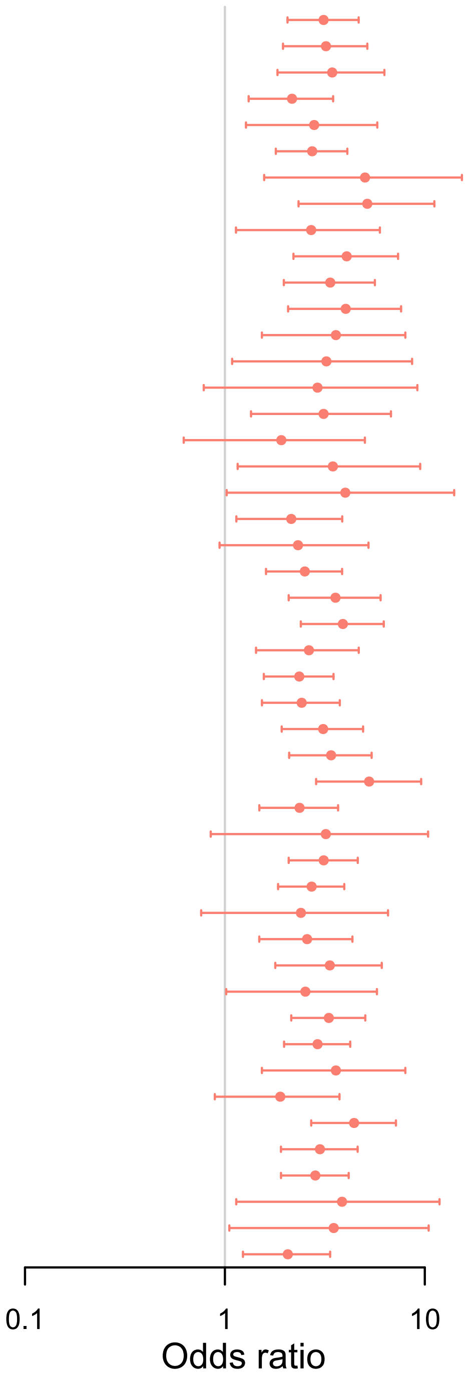

C

Tissue	n	TP / FP	OR	Adj p-value
Adipose Subcutaneous	385	44 / 113	2.81	2.60e-05
Adipose Visceral Omentum	313	32 / 75	2.97	0.000299
Adrenal Gland	175	19 / 39	3.26	0.005916
Artery Aorta	267	28 / 92	2.05	0.160141
Artery Coronary	152	12 / 30	2.61	0.477339
Artery Tibial	388	41 / 117	2.48	0.000676
Brain Amygdala	88	7 / 9	5.02	0.150620
Brain Anterior cingulate cortex BA24	109	15 / 20	4.99	0.000870
Brain Caudate basal ganglia	144	11 / 26	2.75	0.578602
Brain Cerebellar Hemisphere	125	22 / 39	3.83	0.000284
Brain Cerebellum	154	27 / 57	3.26	0.000443
Brain Cortex	136	19 / 32	3.99	0.000746
Brain Frontal Cortex BA9	118	12 / 23	3.42	0.070736
Brain Hippocampus	111	8 / 15	3.45	0.403529
Brain Hypothalamus	108	5 / 11	2.91	1.000
Brain Nucleus accumbens basal ganglia	130	11 / 24	2.98	0.241568
Brain Putamen basal ganglia	111	7 / 21	2.14	1.000
Brain Spinal cord cervical c-1	83	8 / 14	3.70	0.297560
Brain Substantia nigra	80	5 / 9	3.56	1.000
Breast Mammary Tissue	251	17 / 55	2.03	0.997386
Cells EBV-transformed lymphocytes	117	9 / 29	2.00	1.000
Cells Transformed fibroblasts	300	37 / 105	2.46	0.001601
Colon Sigmoid	203	26 / 52	3.43	0.000211
Colon Transverse	246	34 / 65	3.71	2.46e-06
Esophagus Gastroesophageal Junction	213	19 / 54	2.33	0.212216
Esophagus Mucosa	358	43 / 139	2.18	0.006231
Esophagus Muscularis	335	33 / 99	2.29	0.013803
Heart Atrial Appendage	264	33 / 76	3.04	0.000170
Heart Left Ventricle	272	33 / 71	3.26	4.03e-05
Liver	153	23 / 32	4.93	7.43e-06
Lung	383	32 / 101	2.16	0.040382
Minor Salivary Gland	85	6 / 11	3.50	0.983049
Muscle Skeletal	491	48 / 117	3.02	1.60e-06
Nerve Tibial	361	52 / 146	2.62	1.97e-05
Ovary	122	6 / 17	2.26	1.000
Pancreas	220	25 / 63	2.69	0.008274
Pituitary	157	21 / 39	3.64	0.000807
Prostate	132	10 / 24	2.70	0.894255
Skin Not Sun Exposed Suprapubic	335	42 / 96	3.16	3.74e-06
Skin Sun Exposed Lower leg	414	54 / 141	2.86	1.26e-06
Small Intestine Terminal Ileum	122	11 / 23	3.12	0.187878
Spleen	146	12 / 43	1.80	1.000
Stomach	237	35 / 58	4.32	9.00e-08
Testis	225	39 / 91	3.06	1.76e-05
Thyroid	399	49 / 132	2.72	2.10e-05
Uterus	101	6 / 11	3.50	0.983049
Vagina	106	7 / 12	3.76	0.460760
Whole Blood	369	27 / 89	2.04	0.150785

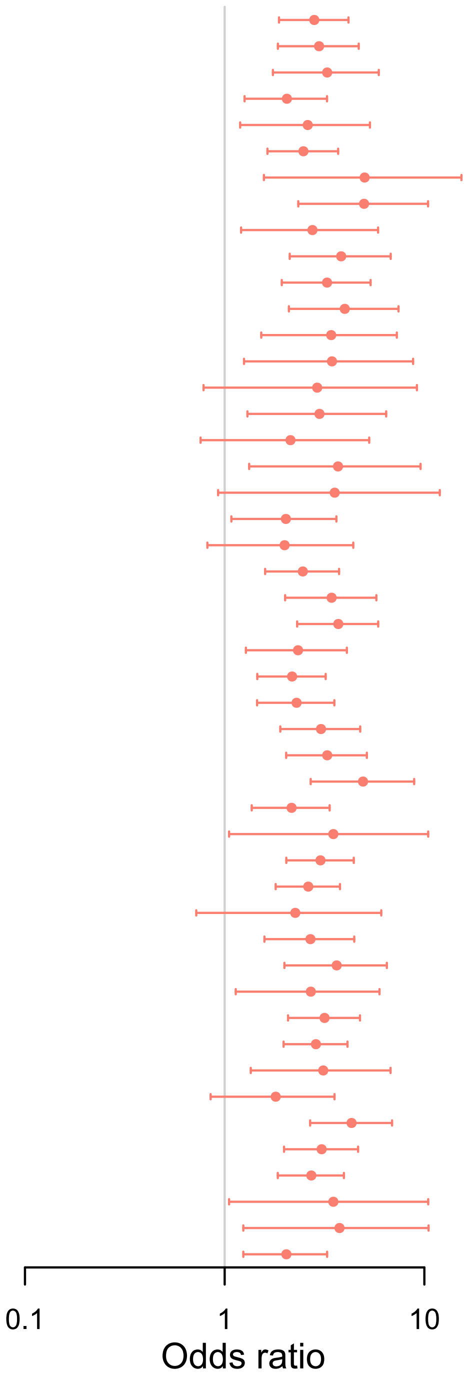

Supplementary Figure 5. Comparison of tissue-specific cis-eQTL data from GTEx v7 (n=48 tissues) for distinguishing true from false positive causal genes at 227 independent mQTLs. Odds ratios and 95% confidence intervals are indicated for cis-eQTL targets of (**a**) sentinel variants, (**b**) proxy variants, and (**c**) either sentinel or proxy variants. The background gene set comprised of all candidate causal genes (both top-down and bottom-up) highlighted by ProGeM. Fisher's exact test was used throughout, and Bonferroni corrected (48 tests) *p*-values are indicated. The number of true positive (TP) and false positive (FP) causal genes identified by each characteristic, as well as the number of samples for each tissue assayed by the GTEx consortium, are also indicated.

a

Tissue	n	TP / FP	OR	Adj p-value
Adipose Subcutaneous	385	37 / 83	1.94	0.1171
Adipose Visceral Omentum	313	26 / 58	1.90	0.7141
Adrenal Gland	175	17 / 32	2.22	0.7589
Artery Aorta	267	24 / 71	1.40	1.000
Artery Coronary	152	7 / 20	1.41	1.000
Artery Tibial	388	35 / 92	1.62	1.000
Brain Amygdala	88	6 / 6	4.08	0.9026
Brain Anterior cingulate cortex BA24	109	13 / 16	3.39	0.0894
Brain Caudate basal ganglia	144	10 / 21	1.95	1.000
Brain Cerebellar Hemisphere	125	17 / 26	2.75	0.1321
Brain Cerebellum	154	22 / 39	2.40	0.1249
Brain Cortex	136	14 / 23	2.53	0.5065
Brain Frontal Cortex BA9	118	10 / 15	2.74	0.9463
Brain Hippocampus	111	7 / 12	2.38	1.000
Brain Hypothalamus	108	5 / 10	2.02	1.000
Brain Nucleus accumbens basal ganglia	130	10 / 13	3.17	0.6636
Brain Putamen basal ganglia	111	7 / 16	1.77	1.000
Brain Spinal cord cervical c-1	83	6 / 10	2.44	1.000
Brain Substantia nigra	80	4 / 8	2.02	1.000
Breast Mammary Tissue	251	14 / 42	1.36	1.000
Cells EBV-transformed lymphocytes	117	5 / 19	1.05	1.000
Cells Transformed fibroblasts	300	33 / 82	1.72	0.8928
Colon Sigmoid	203	21 / 39	2.28	0.2193
Colon Transverse	246	27 / 47	2.48	0.0353
Esophagus Gastroesophageal Junction	213	19 / 46	1.71	1.000
Esophagus Mucosa	358	41 / 105	1.69	0.5054
Esophagus Muscularis	335	32 / 73	1.88	0.3258
Heart Atrial Appendage	264	27 / 52	2.23	0.0948
Heart Left Ventricle	272	28 / 56	2.15	0.1259
Liver	153	17 / 26	2.75	0.1321
Lung	383	28 / 82	1.42	1.000
Minor Salivary Gland	85	4 / 7	2.31	1.000
Muscle Skeletal	491	41 / 90	2.01	0.0490
Nerve Tibial	361	45 / 116	1.70	0.3661
Ovary	122	4 / 12	1.34	1.000
Pancreas	220	20 / 45	1.86	1.000
Pituitary	157	18 / 23	3.32	0.0202
Prostate	132	7 / 22	1.28	1.000
Skin Not Sun Exposed Suprapubic	335	35 / 76	2.00	0.1167
Skin Sun Exposed Lower leg	414	48 / 107	2.02	0.0223
Small Intestine Terminal Ileum	122	7 / 17	1.67	1.000
Spleen	146	9 / 31	1.17	1.000
Stomach	237	24 / 40	2.57	0.0477
Testis	225	31 / 64	2.09	0.1348
Thyroid	399	39 / 98	1.72	0.5533
Uterus	101	5 / 5	4.06	1.000
Vagina	106	5 / 11	1.84	1.000
Whole Blood	369	22 / 67	1.35	1.000

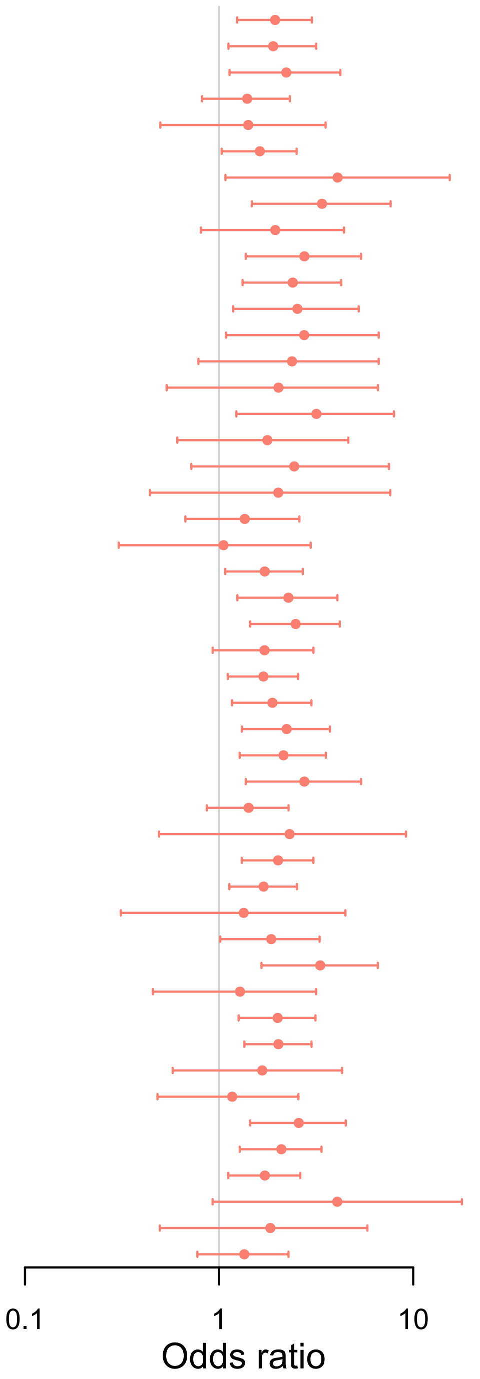

b

Tissue	n	TP / FP	OR	Adj p-value
Adipose Subcutaneous	385	43 / 100	1.90	0.10905
Adipose Visceral Omentum	313	30 / 65	1.98	0.22337
Adrenal Gland	175	19 / 37	2.15	0.71501
Artery Aorta	267	27 / 84	1.33	1.000
Artery Coronary	152	12 / 28	1.76	1.000
Artery Tibial	388	41 / 107	1.66	0.70801
Brain Amygdala	88	7 / 9	3.18	1.000
Brain Anterior cingulate cortex BA24	109	14 / 18	3.25	0.11344
Brain Caudate basal ganglia	144	10 / 24	1.70	1.000
Brain Cerebellar Hemisphere	125	21 / 35	2.55	0.08223
Brain Cerebellum	154	26 / 53	2.09	0.24251
Brain Cortex	136	18 / 30	2.52	0.22375
Brain Frontal Cortex BA9	118	11 / 20	2.26	1.000
Brain Hippocampus	111	7 / 14	2.03	1.000
Brain Hypothalamus	108	5 / 11	1.84	1.000
Brain Nucleus accumbens basal ganglia	130	11 / 23	1.96	1.000
Brain Putamen basal ganglia	111	6 / 20	1.21	1.000
Brain Spinal cord cervical c-1	83	7 / 13	2.19	1.000
Brain Substantia nigra	80	5 / 8	2.53	1.000
Breast Mammary Tissue	251	17 / 52	1.33	1.000
Cells EBV-transformed lymphocytes	117	9 / 25	1.46	1.000
Cells Transformed fibroblasts	300	35 / 97	1.53	1.000
Colon Sigmoid	203	26 / 50	2.23	0.12502
Colon Transverse	246	33 / 60	2.41	0.01623
Esophagus Gastroesophageal Junction	213	19 / 48	1.64	1.000
Esophagus Mucosa	358	42 / 126	1.41	1.000
Esophagus Muscularis	335	33 / 94	1.48	1.000
Heart Atrial Appendage	264	32 / 72	1.91	0.30545
Heart Left Ventricle	272	32 / 66	2.10	0.09970
Liver	153	23 / 30	3.31	0.00307
Lung	383	32 / 93	1.44	1.000
Minor Salivary Gland	85	5 / 10	2.02	1.000
Muscle Skeletal	491	46 / 108	1.89	0.07307
Nerve Tibial	361	49 / 132	1.63	0.52324
Ovary	122	6 / 16	1.51	1.000
Pancreas	220	23 / 60	1.60	1.000
Pituitary	157	19 / 38	2.09	0.76557
Prostate	132	9 / 23	1.59	1.000
Skin Not Sun Exposed Suprapubic	335	40 / 87	2.03	0.06190
Skin Sun Exposed Lower leg	414	50 / 127	1.75	0.18626
Small Intestine Terminal Ileum	122	11 / 20	2.26	1.000
Spleen	146	12 / 41	1.18	1.000
Stomach	237	33 / 53	2.76	0.00166
Testis	225	36 / 85	1.83	0.36262
Thyroid	399	47 / 121	1.71	0.30238
Uterus	101	6 / 10	2.44	1.000
Vagina	106	6 / 11	2.21	1.000
Whole Blood	369	25 / 81	1.26	1.000

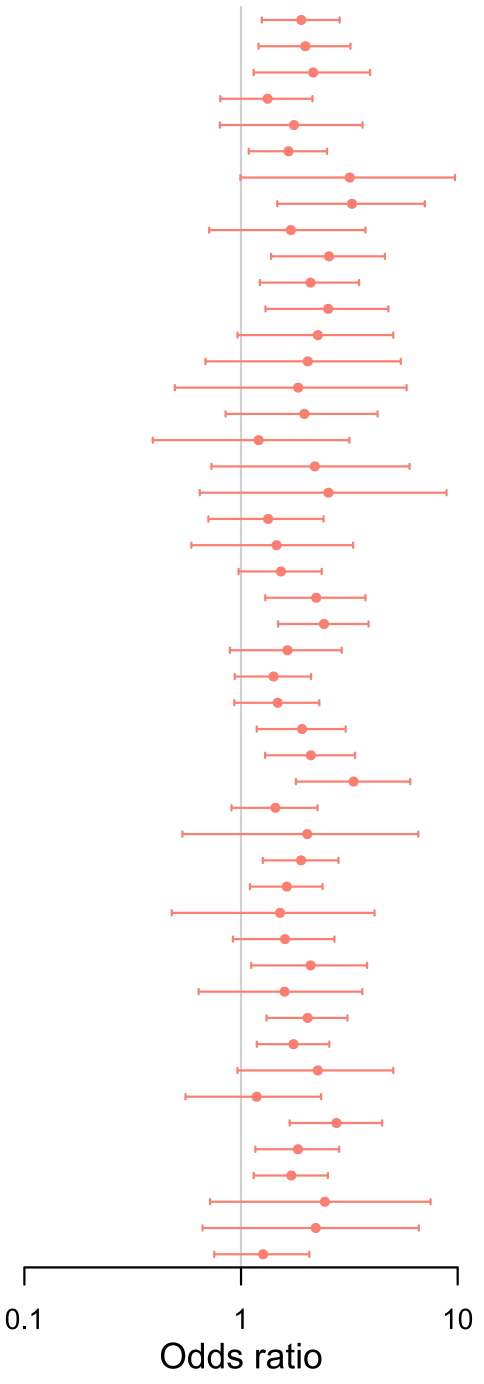

C

Tissue	n	TP / FP	OR	Adj p-value
Adipose Subcutaneous	385	47 / 219	0.82	1.000
Adipose Visceral Omentum	313	32 / 154	0.80	1.000
Adrenal Gland	175	19 / 84	0.90	1.000
Artery Aorta	267	32 / 172	0.70	1.000
Artery Coronary	152	9 / 56	0.63	1.000
Artery Tibial	388	44 / 224	0.73	1.000
Brain Amygdala	88	7 / 19	1.49	1.000
Brain Anterior cingulate cortex BA24	109	13 / 46	1.14	1.000
Brain Caudate basal ganglia	144	10 / 51	0.77	1.000
Brain Cerebellar Hemisphere	125	20 / 74	1.09	1.000
Brain Cerebellum	154	25 / 112	0.88	1.000
Brain Cortex	136	15 / 71	0.83	1.000
Brain Frontal Cortex BA9	118	11 / 44	1.00	1.000
Brain Hippocampus	111	8 / 29	1.11	1.000
Brain Hypothalamus	108	5 / 26	0.76	1.000
Brain Nucleus accumbens basal ganglia	130	11 / 47	0.93	1.000
Brain Putamen basal ganglia	111	8 / 35	0.91	1.000
Brain Spinal cord cervical c-1	83	8 / 27	1.19	1.000
Brain Substantia nigra	80	6 / 18	1.34	1.000
Breast Mammary Tissue	251	19 / 107	0.68	1.000
Cells EBV-transformed lymphocytes	117	9 / 50	0.71	1.000
Cells Transformed fibroblasts	300	39 / 210	0.69	1.000
Colon Sigmoid	203	27 / 107	1.01	1.000
Colon Transverse	246	40 / 135	1.23	1.000
Esophagus Gastroesophageal Junction	213	20 / 110	0.70	1.000
Esophagus Mucosa	358	48 / 273	0.62	0.366
Esophagus Muscularis	335	36 / 183	0.74	1.000
Heart Atrial Appendage	264	36 / 143	1.01	1.000
Heart Left Ventricle	272	33 / 145	0.89	1.000
Liver	153	22 / 83	1.07	1.000
Lung	383	32 / 209	0.55	0.123
Minor Salivary Gland	85	6 / 20	1.21	1.000
Muscle Skeletal	491	48 / 239	0.75	1.000
Nerve Tibial	361	56 / 282	0.72	1.000
Ovary	122	6 / 32	0.74	1.000
Pancreas	220	27 / 136	0.76	1.000
Pituitary	157	22 / 77	1.16	1.000
Prostate	132	10 / 51	0.77	1.000
Skin Not Sun Exposed Suprapubic	335	42 / 207	0.77	1.000
Skin Sun Exposed Lower leg	414	59 / 282	0.78	1.000
Small Intestine Terminal Ileum	122	7 / 52	0.52	1.000
Spleen	146	12 / 82	0.56	1.000
Stomach	237	35 / 115	1.26	1.000
Testis	225	37 / 182	0.77	1.000
Thyroid	399	49 / 258	0.69	1.000
Uterus	101	6 / 17	1.42	1.000
Vagina	106	7 / 23	1.22	1.000
Whole Blood	369	28 / 180	0.57	0.432

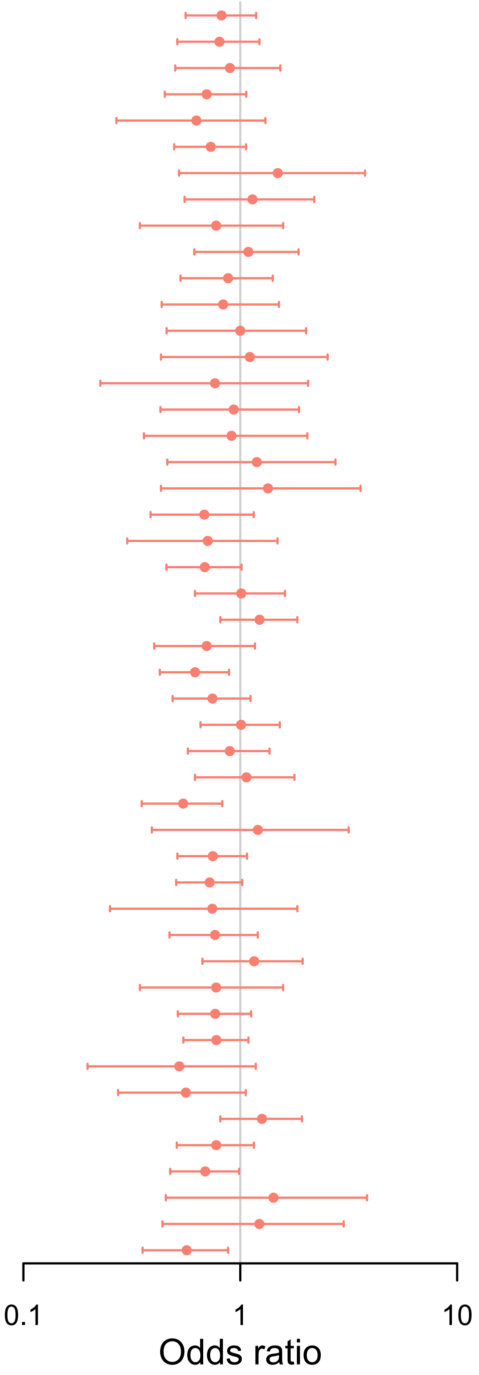

Supplementary Figure 6. Comparison of tissue-specific cis-eQTL data from GTEx v7 (n=48 tissues) for distinguishing true from false positive causal genes at 227 independent mQTLs using the bottom-up background gene set. Odds ratios and 95% confidence intervals are indicated for cis-eQTL targets of (**a**) sentinel variants, (**b**) proxy variants, and (**c**) either sentinel or proxy variants are indicated. The background gene set comprised of all bottom-up candidate causal genes highlighted by ProGeM. Fisher's exact test was used throughout, and Bonferroni corrected (48 tests) *p*-values are indicated. The number of true positive (TP) and false positive (FP) causal genes identified by each characteristic, as well as the number of samples for each tissue assayed by the GTEx consortium, are also indicated.

a

Tissue	n	TP / FP	OR	Adj p-value
Adipose Subcutaneous	385	99 / 118	3.52	3.80e-15
Adipose Visceral Omentum	313	72 / 84	3.45	6.09e-11
Adrenal Gland	175	44 / 42	4.07	3.20e-08
Artery Aorta	267	73 / 76	3.89	8.52e-13
Artery Coronary	152	23 / 23	3.76	0.000750
Artery Tibial	388	93 / 128	2.99	2.53e-11
Brain Amygdala	88	17 / 4	15.94	4.45e-07
Brain Anterior cingulate cortex BA24	109	18 / 24	2.79	0.088970
Brain Caudate basal ganglia	144	29 / 27	4.08	2.17e-05
Brain Cerebellar Hemisphere	125	29 / 31	3.55	0.000157
Brain Cerebellum	154	43 / 58	2.85	6.67e-05
Brain Cortex	136	29 / 33	3.33	0.000531
Brain Frontal Cortex BA9	118	28 / 22	4.84	3.83e-06
Brain Hippocampus	111	18 / 13	5.18	0.000441
Brain Hypothalamus	108	19 / 9	7.93	7.44e-06
Brain Nucleus accumbens basal ganglia	130	24 / 27	3.34	0.001964
Brain Putamen basal ganglia	111	20 / 17	4.41	0.000610
Brain Spinal cord cervical c-1	83	14 / 14	3.71	0.036623
Brain Substantia nigra	80	10 / 7	5.28	0.042032
Breast Mammary Tissue	251	50 / 55	3.55	6.15e-08
Cells EBV-transformed lymphocytes	117	26 / 15	6.58	3.04e-07
Cells Transformed fibroblasts	300	76 / 103	2.97	2.42e-09
Colon Sigmoid	203	46 / 53	3.36	1.16e-06
Colon Transverse	246	61 / 48	5.09	4.39e-14
Esophagus Gastroesophageal Junction	213	53 / 62	3.35	8.47e-08
Esophagus Mucosa	358	93 / 125	3.07	8.72e-12
Esophagus Muscularis	335	83 / 118	2.85	1.96e-09
Heart Atrial Appendage	264	62 / 68	3.62	6.92e-10
Heart Left Ventricle	272	53 / 62	3.35	8.47e-08
Liver	153	28 / 31	3.42	0.000347
Lung	383	90 / 106	3.51	7.48e-14
Minor Salivary Gland	85	15 / 12	4.66	0.005100
Muscle Skeletal	491	88 / 117	3.08	2.75e-11
Nerve Tibial	361	112 / 151	3.15	1.64e-14
Ovary	122	16 / 22	2.70	0.206036
Pancreas	220	58 / 64	3.58	2.52e-09
Pituitary	157	32 / 47	2.57	0.005906
Prostate	132	19 / 22	3.22	0.017165
Skin Not Sun Exposed Suprapubic	335	70 / 95	2.94	2.06e-08
Skin Sun Exposed Lower leg	414	105 / 144	3.06	4.04e-13
Small Intestine Terminal Ileum	122	28 / 22	4.84	3.83e-06
Spleen	146	47 / 35	5.26	3.44e-11
Stomach	237	51 / 49	4.08	2.27e-09
Testis	225	58 / 97	2.32	0.000130
Thyroid	399	93 / 151	2.50	2.03e-08
Uterus	101	14 / 11	4.73	0.007789
Vagina	106	13 / 10	4.83	0.011889
Whole Blood	369	82 / 112	2.97	4.96e-10

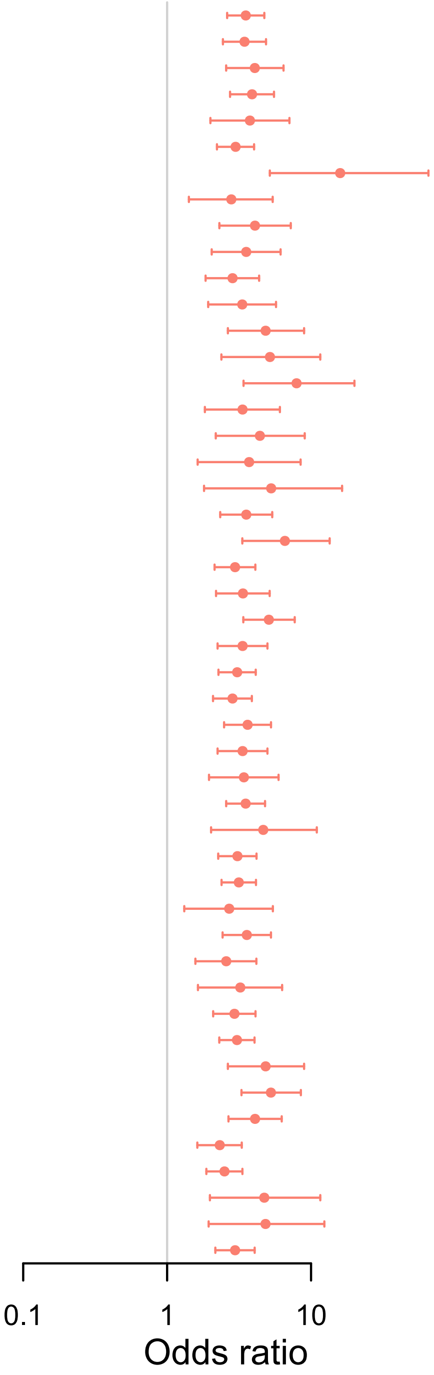

b

Tissue	n	TP / FP	OR	Adj p-value
Adipose Subcutaneous	385	106 / 173	2.53	1.16e-09
Adipose Visceral Omentum	313	83 / 122	2.75	6.09e-09
Adrenal Gland	175	52 / 71	2.85	5.29e-06
Artery Aorta	267	82 / 119	2.78	6.99e-09
Artery Coronary	152	34 / 37	3.51	2.58e-05
Artery Tibial	388	109 / 183	2.47	1.89e-09
Brain Amygdala	88	17 / 13	4.89	0.001228
Brain Anterior cingulate cortex BA24	109	19 / 38	1.85	1.000
Brain Caudate basal ganglia	144	36 / 48	2.86	0.000425
Brain Cerebellar Hemisphere	125	30 / 52	2.17	0.071318
Brain Cerebellum	154	47 / 83	2.17	0.004878
Brain Cortex	136	37 / 58	2.42	0.004809
Brain Frontal Cortex BA9	118	35 / 40	3.34	4.64e-05
Brain Hippocampus	111	19 / 20	3.55	0.010043
Brain Hypothalamus	108	25 / 27	3.49	0.000907
Brain Nucleus accumbens basal ganglia	130	27 / 43	2.36	0.047567
Brain Putamen basal ganglia	111	27 / 32	3.18	0.001528
Brain Spinal cord cervical c-1	83	17 / 19	3.33	0.030984
Brain Substantia nigra	80	12 / 9	4.94	0.018134
Breast Mammary Tissue	251	57 / 76	2.94	6.40e-07
Cells EBV-transformed lymphocytes	117	26 / 35	2.79	0.009312
Cells Transformed fibroblasts	300	90 / 139	2.63	5.78e-09
Colon Sigmoid	203	56 / 82	2.66	8.69e-06
Colon Transverse	246	71 / 75	3.82	3.12e-12
Esophagus Gastroesophageal Junction	213	61 / 85	2.82	8.15e-07
Esophagus Mucosa	358	98 / 181	2.19	1.38e-06
Esophagus Muscularis	335	91 / 170	2.14	8.14e-06
Heart Atrial Appendage	264	68 / 102	2.64	8.80e-07
Heart Left Ventricle	272	63 / 91	2.72	1.07e-06
Liver	153	33 / 40	3.14	0.000201
Lung	383	97 / 162	2.44	3.38e-08
Minor Salivary Gland	85	18 / 15	4.49	0.001428
Muscle Skeletal	491	90 / 168	2.14	7.49e-06
Nerve Tibial	361	126 / 222	2.39	4.19e-10
Ovary	122	18 / 27	2.48	0.243245
Pancreas	220	68 / 94	2.87	5.90e-08
Pituitary	157	38 / 62	2.33	0.007288
Prostate	132	27 / 33	3.09	0.001939
Skin Not Sun Exposed Suprapubic	335	89 / 145	2.48	7.73e-08
Skin Sun Exposed Lower leg	414	115 / 200	2.39	2.91e-09
Small Intestine Terminal Ileum	122	35 / 29	4.63	1.98e-07
Spleen	146	62 / 64	3.86	1.10e-10
Stomach	237	56 / 68	3.23	7.87e-08
Testis	225	68 / 153	1.71	0.037622
Thyroid	399	108 / 224	1.95	2.97e-05
Uterus	101	15 / 20	2.78	0.268369
Vagina	106	16 / 16	3.72	0.015364
Whole Blood	369	98 / 170	2.35	1.07e-07

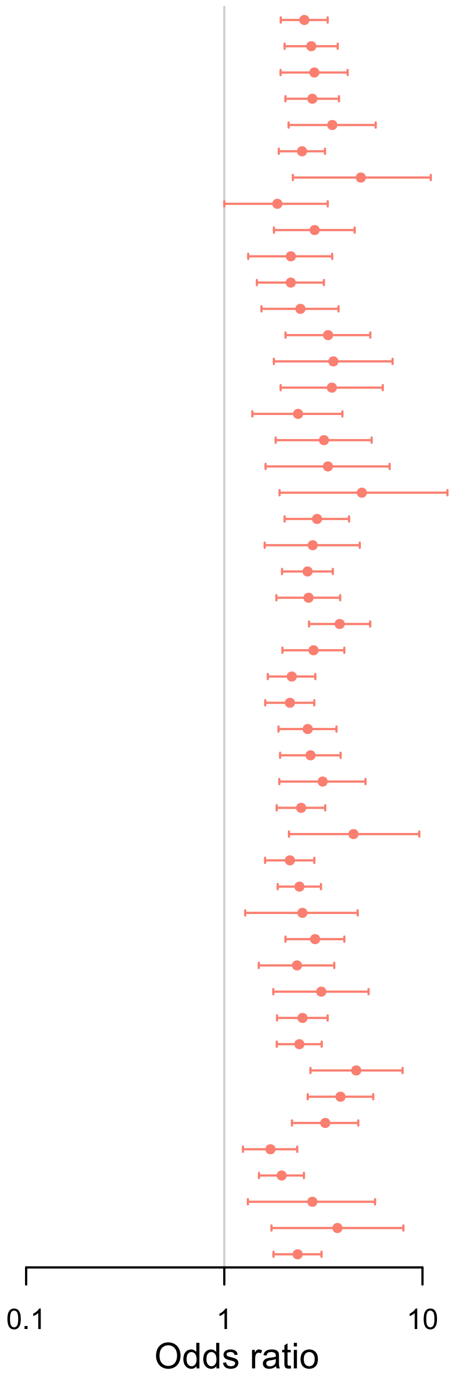

C

Tissue	n	TP / FP	OR	Adj p-value
Adipose Subcutaneous	385	114 / 178	2.69	1.85e-11
Adipose Visceral Omentum	313	86 / 124	2.82	1.45e-09
Adrenal Gland	175	54 / 73	2.88	2.28e-06
Artery Aorta	267	87 / 121	2.93	3.07e-10
Artery Coronary	152	35 / 37	3.62	1.13e-05
Artery Tibial	388	115 / 185	2.61	6.49e-11
Brain Amygdala	88	18 / 13	5.18	0.000441
Brain Anterior cingulate cortex BA24	109	20 / 39	1.90	1.000
Brain Caudate basal ganglia	144	37 / 49	2.88	0.000280
Brain Cerebellar Hemisphere	125	31 / 55	2.12	0.089684
Brain Cerebellum	154	51 / 86	2.28	0.000716
Brain Cortex	136	39 / 58	2.56	0.001064
Brain Frontal Cortex BA9	118	36 / 40	3.44	2.36e-05
Brain Hippocampus	111	19 / 21	3.38	0.012766
Brain Hypothalamus	108	26 / 28	3.50	0.000585
Brain Nucleus accumbens basal ganglia	130	29 / 44	2.48	0.019205
Brain Putamen basal ganglia	111	28 / 32	3.31	0.000827
Brain Spinal cord cervical c-1	83	17 / 19	3.33	0.030984
Brain Substantia nigra	80	12 / 9	4.94	0.018134
Breast Mammary Tissue	251	58 / 80	2.84	1.19e-06
Cells EBV-transformed lymphocytes	117	27 / 35	2.91	0.005218
Cells Transformed fibroblasts	300	94 / 147	2.61	4.69e-09
Colon Sigmoid	203	58 / 84	2.70	3.84e-06
Colon Transverse	246	75 / 77	3.96	2.41e-13
Esophagus Gastroesophageal Junction	213	64 / 87	2.91	1.17e-07
Esophagus Mucosa	358	106 / 185	2.35	2.31e-08
Esophagus Muscularis	335	99 / 174	2.32	1.47e-07
Heart Atrial Appendage	264	74 / 104	2.85	1.57e-08
Heart Left Ventricle	272	66 / 92	2.84	1.38e-07
Liver	153	33 / 43	2.91	0.000628
Lung	383	101 / 165	2.51	3.98e-09
Minor Salivary Gland	85	18 / 17	3.96	0.004028
Muscle Skeletal	491	96 / 170	2.29	4.10e-07
Nerve Tibial	361	136 / 229	2.56	3.37e-12
Ovary	122	19 / 28	2.53	0.163038
Pancreas	220	70 / 98	2.84	5.78e-08
Pituitary	157	40 / 65	2.34	0.003861
Prostate	132	28 / 37	2.85	0.004046
Skin Not Sun Exposed Suprapubic	335	91 / 152	2.42	9.74e-08
Skin Sun Exposed Lower leg	414	122 / 212	2.42	7.25e-10
Small Intestine Terminal Ileum	122	36 / 30	4.61	1.29e-07
Spleen	146	63 / 64	3.93	2.87e-11
Stomach	237	58 / 70	3.26	3.25e-08
Testis	225	73 / 153	1.85	0.004064
Thyroid	399	114 / 230	2.02	3.08e-06
Uterus	101	16 / 20	2.97	0.085547
Vagina	106	17 / 17	3.73	0.009966
Whole Blood	369	106 / 175	2.50	2.28e-09

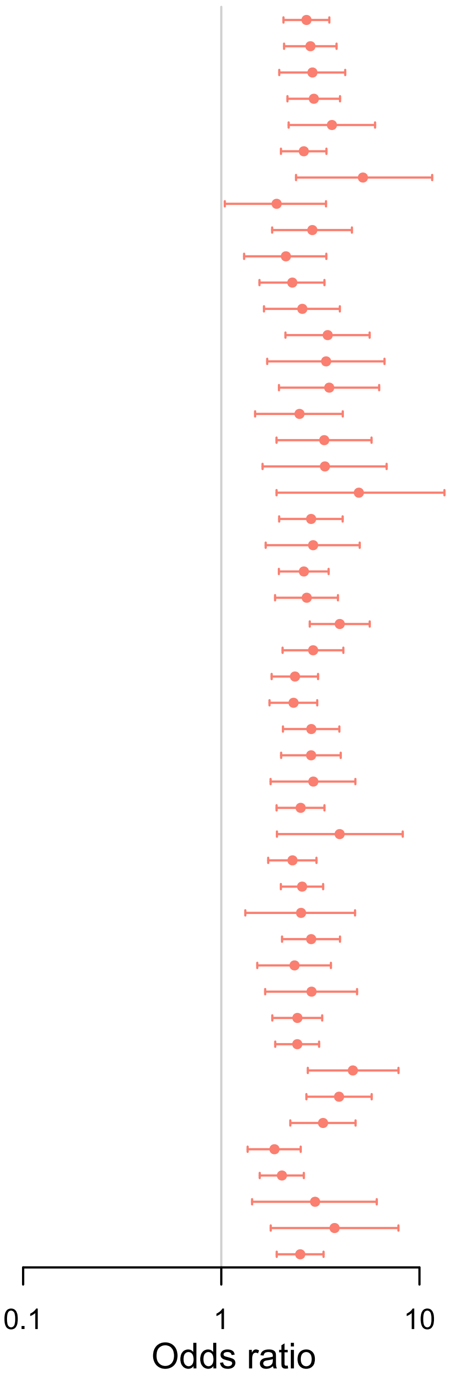

Supplementary Figure 7. Comparison of tissue-specific cis-eQTL from GTEx v7 data (n=48 tissues) for distinguishing true from false positive causal genes at 562 independent cis-pQTLs. Odds ratios and 95% confidence intervals are indicated for cis-eQTL targets of (**a**) sentinel variants, (**b**) proxy variants, and (**c**) either sentinel or proxy variants are indicated. The background gene set comprised of all bottom-up candidate causal genes highlighted by ProGeM. Fisher's exact test was used throughout, and Bonferroni corrected (48 tests) *p*-values are indicated. The number of true positive (TP) and false positive (FP) causal genes identified by each characteristic, as well as the number of samples for each tissue assayed by the GTEx consortium, are also indicated.
